# Distress severity in perceptual anomalies moderates the relationship between prefrontal brain structure and psychosis proneness in nonclinical individuals

**DOI:** 10.1007/s00406-020-01229-5

**Published:** 2021-02-02

**Authors:** Ulrika Evermann, Simon Schmitt, Tina Meller, Julia-Katharina Pfarr, Sarah Grezellschak, Igor Nenadić

**Affiliations:** 1 Cognitive Neuropsychiatry Lab, Department of Psychiatry and Psychotherapy, Philipps-Universität Marburg, Rudolf-Bultmann-Str. 8, 35039 Marburg, Germany; 2 Center for Mind, Brain and Behavior (CMBB), Hans-Meerwein-Str. 6, 35032 Marburg, Germany; 3 Marburg University Hospital-UKGM, Rudolf-Bultmann-Str. 8, 35039 Marburg, Germany

**Keywords:** Psychological distress, Psychotic-like experiences, Prodromal questionnaire, Psychosis risk

## Abstract

In the general population, psychosis risk phenotypes occur independently of attenuated prodromal syndromes. Neurobiological correlates of vulnerability could help to understand their meaningfulness. Interactions between the occurrence of psychotic-like experiences (PLE) and other psychological factors e.g., distress related to PLE, may distinguish psychosis-prone individuals from those without risk of future psychotic disorder. We aimed to investigate whether (a) correlates of total PLE and distress, and (b) symptom dimension-specific moderation effects exist at the brain structural level in non-help-seeking adults reporting PLE below and above the screening criterion for clinical high-risk (CHR). We obtained T1-weighted whole-brain MRI scans from 104 healthy adults from the community without psychosis CHR states for voxel-based morphometry (VBM). Brain structural associations with PLE and PLE distress were analysed with multiple linear regression models. Moderation of PLE by distress severity of two types of positive symptoms from the Prodromal Questionnaire (PQ-16) screening inventory was explored in regions-of-interest after VBM. Total PQ-16 score was positively associated with grey matter volume (GMV) in prefrontal regions, occipital fusiform and lingual gyri (*p* < 0.05, FDR peak-level corrected). Overall distress severity and GMV were not associated. Examination of distress severity on the positive symptom dimensions as moderators showed reduced strength of the association between PLE and rSFG volume with increased distress severity for perceptual PLE. In this study, brain structural variation was related to PLE level, but not distress severity, suggesting specificity. In healthy individuals, positive relationships between PLE and prefrontal volumes may indicate protective features, which supports the insufficiency of PLE for the prediction of CHR. Additional indicators of vulnerability, such as distress associated with perceptual PLE, change the positive brain structure relationship. Brain structural findings may strengthen clinical objectives through disentanglement of innocuous and risk-related PLE.

## Introduction

Prevention of psychosis spectrum disorders relies on early risk detection [1]. Prediction of transition to psychosis is particularly enhanced when clinically validated assessments are employed in targeted samples found in specialised mental health services [2]. On the other hand, the use of instruments to assess clinical high risk (CHR) states in general non-help-seeking populations produces weak predictive estimates of the true risk for imminent psychosis [3]. This shortcoming has been encountered by psychometric developments building on two-staged assessments of psychosis CHR states by screening and semi-structured clinical interviews, which enables improved clinical efficiency and accuracy [4]. Originally validated in a general mental health help-seeking population, the abbreviated 16-item version of the Prodromal Questionnaire [5] (PQ-16) sufficiently screens for psychosis ultra-high risk (UHR) states [6]. Together the Prodromal Questionnaires (92, 21, and 16-item versions) [5–7] are among the most widely used CHR screening tools [8]. Previous studies have employed the PQ-16 among help-seeking adults [9] and adolescents [10], as well as nonclinical populations [11, 12] for a review see Ref. [13].

The prevalence of subclinical psychotic experiences exceeds that of psychosis in the general population [14], but self-reported psychotic-like experiences (PLE) themselves constitute an inadequate criterion for attenuated psychotic syndromes [15]. Besides clinical prodromal symptoms, screening inventories such as PQ-16, therefore, capture PLE in a broader perspective. Among CHR individuals, motivation to seek help for distressful prodromal symptoms is increased by the burden of affective symptoms leading to greater functional decline [16]; these factors are also captured by semi-structured interviews for attenuated psychotic syndromes [17]. In the general population, evidence exists that persistence of PLE [18], distress [19, 20], and emotional context [21], depression, and reduced functioning [22] associated with positive PLE indicate elevated clinical relevance. The importance of distress for the differentiation between PLE with reduced clinical significance as, for instance, in developmental cohorts [23] and attenuated psychosis risk was also reflected in the uptake of an additional distress severity subscale to the prodromal screening inventory [7].

Multiple neuroimaging studies compared brain morphology in ultra-high risk (UHR) for psychosis to healthy controls or first-episode psychosis patients [24–27]. In contrast to case–control brain imaging studies, which have focused on UHR and first-episode psychosis [28], the nonclinical spectrum (i.e., the occurrence of sparse PLE in healthy subjects) has received less attention despite recent findings of dimensional relations on the phenotype level [29, 30]. A continuous relationship between infrequent psychotic-like or subclinical symptoms towards a clinical spectrum [31–33] permits a hypothesised relation to neural markers that have been associated with CHR or disease status. This may add to the current understanding of the brain-behaviour relationships in the psychosis spectrum and the development of biomarkers in the early intervention field. Previous studies report associations between subclinical psychotic experiences and brain volume, as well as functional and cortical surface variation [34, 35], some of which converge with alterations typically found in the manifest psychosis spectrum and affective disorders [36]. Across the literature, PLE are associated with structural change in diverse cortical regions, e.g., orbitofrontal and medial temporal lobes [37] and the parietal regions [38]. However, a strong effect for PLE associated with any particular cortical regions derived by meta-analysis is presently lacking. A recent study from our group showed consistent relationships with volume reductions in prefrontal and anterior cingulate regions across multidimensional schizotypy [39], representing a trait-level schizophrenia endophenotype [40, 41]. Furthermore, the relationship between positive schizotypy and PLE [42–44] is considered to reflect biological psychosis-prone components within schizophrenia endophenotypes [45, 46]. Thus, extending the search for neurobiological correlates relating to PLE may shed further light on the dopaminergic fronto-striatal pathway [47, 48] in nonclinical psychosis phenotypes [39, 49].

Building on previous studies [34, 36, 39], we replicate dimensional approaches using whole-brain voxel-wise analysis. Complementary regional analyses are based on primary outcomes to achieve robust targets relevant to the study cohort. The first aim of this investigation was to examine associations between PLE, PLE distress severity, and brain structure. We predict brain structural reductions in association with subclinical PLE and distress severity. Furthermore, we explored the influence of the interaction of PLE and PLE-related distress severity on regional brain volume.

## Methods

### Sample

A total of 104 participants (71 females, 33 males; mean age = 24.96, SD = 4.76, min = 18, max = 40), all fluent speakers of the German language, were recruited from the local community using advertisements and the university email circulation service. The study protocol adhered to the Declaration of Helsinki [50] and was approved by the local ethics committee of the School of Medicine, Philipps-University of Marburg. Based on an initial telephone screening protocol, we obtained information on exclusion criteria: medical history (neurological or untreated chronic medical condition), past and current substance use, and any history of psychiatric or neurological disorders and treatments including psychotropic medication. Participants aged 18–40 years were then screened using the German version of the Structured Clinical Interview for DSM-IV (SCID-I) [51]. Participants provided written informed consent once invited to complete brain scans and online questionnaires [52], and received financial compensation after participation. Mean laterality quotient of handedness [53] within this cohort was 71.91 (SD = 62.07). An estimated intelligence quotient (IQ) [54] below 80 was exclusionary. The mean IQ estimate was 117.46 (SD = 14.66).

### Prodromal Questionnaire (PQ-16)

**Fig. 1:**
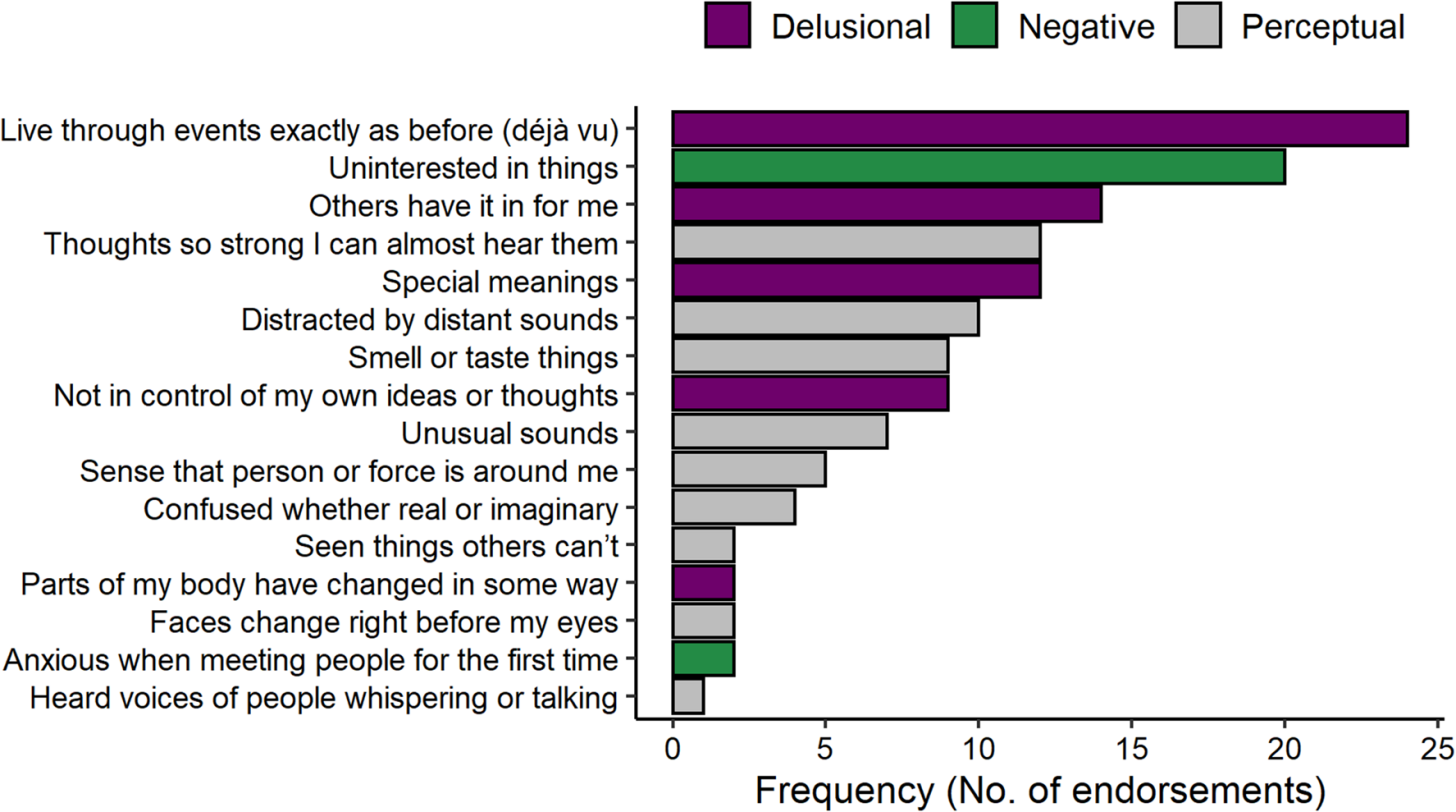
Distribution of psychotic-like experiences (PLE) captured by the German version of the Prodromal Questionnaire 16 (PQ-16) in 104 nonclinical subjects. Most PLE were assigned to three categories reflecting positive (*Delusional, Perceptual*) and *Negative* PLE based on a comparison to the Structured Interview for Prodromal Syndromes (SIPS). *Note*: Item descriptions are abbreviated for display purposes. This figure was created using ggplot2 [94]

We assessed PLE using the 16-item Prodromal Questionnaire (PQ-16) [6], a self-report measure to assess presence of PLE developed from prior versions [5, 7]. The validation of the 16-item version showed that a cutoff of ≥6 endorsed PLE identifies UHR states with 87% sensitivity and 87% specificity [6]. Complementary to the total sum of item endorsements on the 2-point scale (‘true’/’false’), a measure of distress severity for each endorsed item is obtained on a 4-point scale from 0 (‘none’) to 3 (‘severe’). In addition to the total symptom score, the distress severity scale cutoff score ≥9 was recommended in a study of non-help-seeking subjects [55]. Based on previous psychometric studies [5, 6, 23, 56] and guided by item comparison to the German version of the Structured Interview for Prodromal Syndromes Version 5.0 (SIPS) [17], we assigned items to two positive symptom subscales reflecting ‘*Perceptual abnormalities/Hallucinations’* (*Perceptual:* items 3, 4, 5, 6, 8, 9, 12, 13, 15), ‘*Unusual thought content/Delusional ideas’* (*Delusional:* items 2, 10, 11, 14, 16), and *Negative symptoms* (items 1 and 7) (Table 1). Table 2 displays Cronbach’s alpha as measures of internal consistency for these scales, and frequency of single item endorsements within this community sample is shown in Fig. 1.

**Table 1:** Descriptive statistics of PLE in 104 healthy adults assessed by Prodromal Questionnaire (PQ-16)

Prodromal questionnaire (PQ-16)	Total scale		Distress scale			
	Mean	SD^a^	Mean	SD^a^	*r*^b^	*p*_*FDR*_^c^
PLE score	1.30	1.78	1.44	2.15	0.92	< 0.001
*Perceptual abnormalities/Hallucinations*	0.50	1.01	0.56	1.21	0.68	< 0.001
I sometimes smell or taste things that other people can’t smell or taste.	0.09	0.28	0.09	0.34	0.37	< 0.001
I often hear unusual sounds like banging, clicking, hissing, clapping or ringing in my ears.	0.07	0.25	0.08	0.39	0.33	0.001
I have been confused at times whether something I experienced was real or imaginary.	0.04	0.19	0.06	0.31	0.31	0.001
When I look at a person, or look at myself in a mirror, I have seen the face change right before my eyes.	0.02	0.14	0.02	0.14	0.22	0.026
I have seen things that other people apparently can’t see.	0.02	0.14	0.02	0.14	0.24	0.019
My thoughts are sometimes so strong that I can almost hear them.	0.12	0.32	0.14	0.51	0.37	< 0.001
Sometimes I feel suddenly distracted by distant sounds that I am not normally aware of.	0.10	0.30	0.10	0.33	0.39	< 0.001
I have heard things other people can’t hear like voices of people whispering or talking.	0.01	0.10	0.02	0.20	0.15	0.126
I have had the sense that some person or force is around me, even though I could not see anyone.	0.05	0.21	0.04	0.19	0.36	< 0.001
*Unusual thought content/Delusional ideas*	0.59	0.89	0.65	1.10	0.75	< 0.001
I often seem to live through events exactly as they happened before (déjà vu).	0.23	0.42	0.26	0.61	0.47	< 0.001
I sometimes see special meanings in advertisements, shop windows, or in the way things are arranged around me.	0.12	0.32	0.12	0.43	0.48	< 0.001
Sometimes I have felt that I’m not in control of my own ideas or thoughts.	0.09	0.28	0.10	0.38	0.37	< 0.001
I often feel that others have it in for me.	0.13	0.34	0.18	0.50	0.45	< 0.001
I feel that parts of my body have changed in some way, or that parts of my body are working differently than before.	0.02	0.14	0.00	0.00	0.21	0.033
*Negative symptoms*	0.21	0.43	0.23	0.58	0.47	< 0.001
I feel uninterested in the things I used to enjoy.	0.19	0.40	0.21	0.53	0.43	< 0.001
I get extremely anxious when meeting people for the first time.	0.02	0.14	0.02	0.14	0.25	0.015

^a^SD = standard deviation

^b^*r* = Spearman correlation coefficient

^c^*p*_FDR_ = *p-*value after false discovery rate (FDR) adjustment

**Table 2:** Reliability measures for subscales derived from the Prodromal Questionnaire (PQ-16)

PQ-16 scale	Min	Max	Skew	Kurtosis	*α*^a^
Total PLE	0	9	1.84	3.65	0.69
Total PLE distress	0	10	1.77	2.79	0.58
Perceptual scale					
Total	0	5	2.79	8.48	0.62
Distress	0	6	2.61	6.81	0.47
Delusional scale					
Total	0	4	1.51	1.84	0.46
Distress	0	5	1.79	2.88	0.28
Negative scale					
Total	0	2	1.79	2.20	0.13
Distress	0	3	3.01	10.00	0.18

^a^*α* = Cronbach’s alpha

### MRI acquisition and voxel-based morphometry (VBM)

We obtained high-resolution T1-weighted MRI using a 3.0-T Siemens Tim Trio scanner (Siemens, Erlangen, Germany) with standard 12-channel quadrature head coil and a 3D magnetisation-prepared rapid-acquisition gradient echo (MP-RAGE) sequence (4:26 min; TE = 2.26 ms, TI = 900 ms, TR = 1900 ms, 1 mm^3^ isotropic voxel resolution). We then used the Computational Anatomy Toolbox for SPM (CAT12 v12.6, r1450, Christian Gaser, Structural Brain Mapping Group, Jena University Hospital, Germany) in SPM12 (v7219, Statistical Parametric Mapping, Wellcome Trust Centre for Neuroimaging, London, UK) for correction of homogeneity bias and segmentation of T1-weighted images into grey (GM) and white matter and cerebrospinal fluid. All images passed both visual inspection and CAT12 quality assessment protocols. Internal GM threshold was set to 0.1 and scans were smoothed with a full width at half maximum Gaussian kernel of 8 mm.

### Statistical analyses: general linear models

Multiple linear regression models were conducted in SPM12 (v7487) running in Matlab (R2017a, The Mathworks Inc., USA) to test associations between grey matter volume (GMV) and total PLE score and distress severity score, respectively. Age, sex, and total intracranial volume (TIV) were entered as control variables to these models. In these voxel-wise volumetric analyses, the statistical threshold was set to *p* < 0.05 applying false-discovery-rate (FDR) peak-level correction. Anatomical labelling of maximum voxel coordinates was based on the DARTEL neuromorphometrics atlas.

### Moderation analyses

Using the regions-of-interest tool within CAT12.5 (r1363), we extracted estimated mean GMV for each participant based on the neuromorphometrics atlas. These volumes of interest (VOI) were dependent variables in moderation analyses conducted in PROCESS 3.3 [57] for SPSS (Version 25.0, IBM Corp., Armonk, NY). Interactions of Total PLE × distress for *Perceptual* and *Delusional* distress severity were examined as estimators of VOI. Due to the low item and score range, we refrained from including *Negative symptoms* in moderation analyses. We corrected coefficient *p* values for multiple comparisons for the number of dependent variables (VOI) for each PLE subscale using FDR adjusted *p* values. FDR corrections for multiple comparisons [58] were carried out in R [59].

## Results

### PLE screening outcomes

On average, at least one PLE (*M* = 1.30, SD = 1.78, scale score range = 0–9) and a mean distress dimension score of 1.44 (SD = 2.15, scale score range = 0–10) was reported in the present sample. Table 1 provides descriptive statistics for each PQ-16 item, the three PLE subscales and correlations with the overall distress score with two-sided significance levels. Four participants met the clinical screening threshold (PQ-16 total score ≥6 and/or PQ-16 distress score ≥9) and were invited to a follow-up assessment for CHR status using Schizophrenia Proneness Instrument, Adult version (SPI-A) [60]. Three participants completed the clinical interview; none met basic symptom criteria.

### VBM outcomes for PLE

**Fig. 2:**
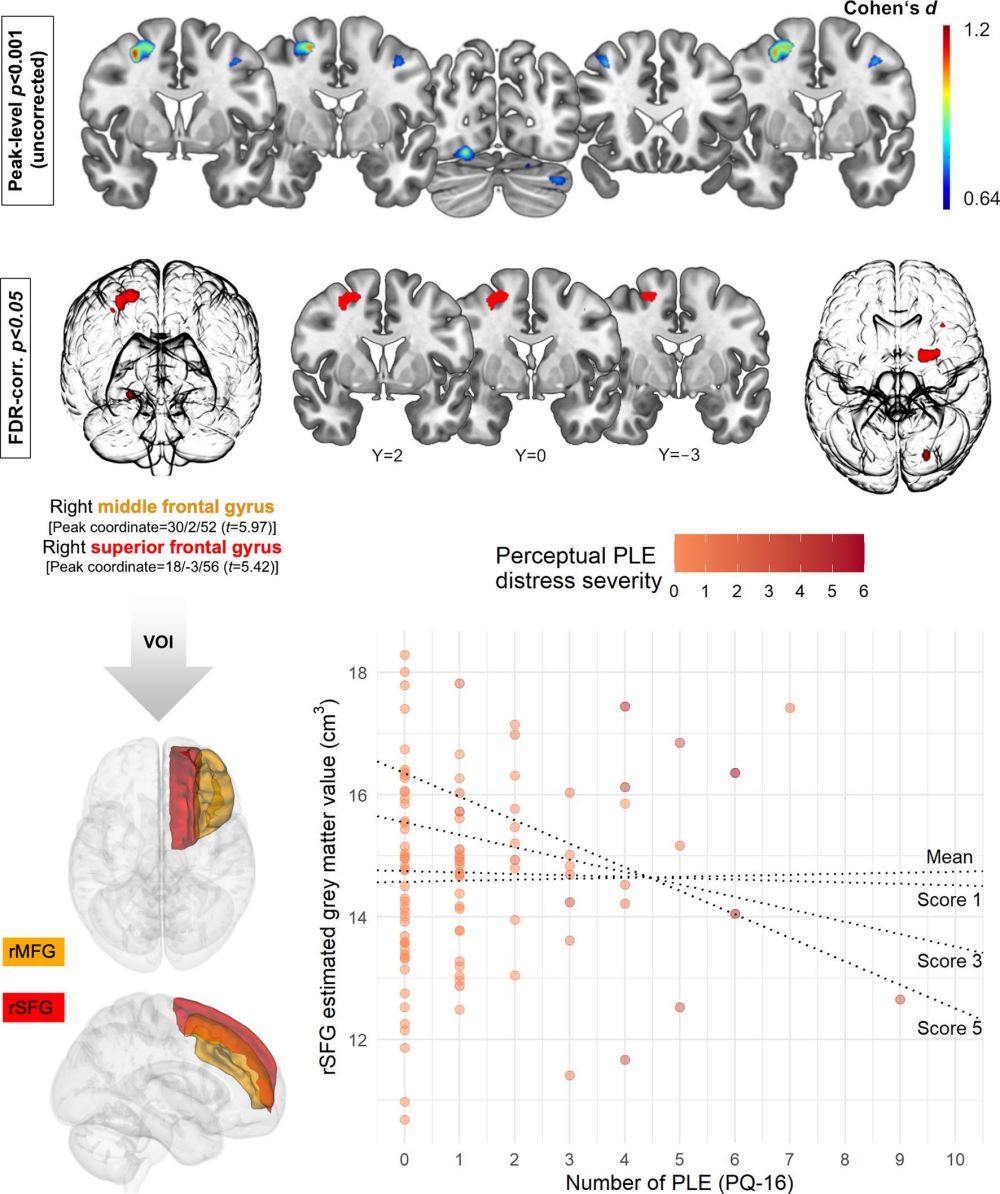
Upper panel shows statistical significance (thresholded at FDR-corrected *p* < 0.05) and effect size (thresholded at uncorrected peak-level *p* < 0.001) maps for structural correlates of total psychotic-like experiences (PLE), assessed by the Prodromal Questionnaire (PQ-16). Mean volumes of interest (VOI) were extracted from two prefrontal regions: right superior (rSFG) and middle frontal gyri (rMFG), which enclose the largest cluster of size *k* = 246. Lower panel shows the effect of distress and PLE interaction on predicted rSFG volume. At higher *Perceptual* PLE distress severity (scale score ≥ 2.75), overall PLE are associated with predicted rSFG volume reductions. This figure was created using MRIcroGL (https://www.mccauslandcenter.sc.edu/mricrogl/), ggplot2 [94], 3D Slicer (https://www.slicer.org) [95] and GNU Image Manipulation Program (GIMP, https://www.gimp.org/)

Total PLE score showed a significant positive association with volume in the right prefrontal region (cluster size *k* = 246) with two significant peaks at the right superior (rSFG, maximum voxel coordinates *X*/*Y*/*Z* = 18/ − 3/56, *t* = 5.42, *p* = 0.009) and middle frontal gyrus (rMFG) (maximum voxel coordinates *X*/*Y*/*Z* = 30/2/52, *t* = 5.97, *p* = 0.005). PQ-16 associations were significant at the FDR-corrected statistical threshold in the occipital fusiform and lingual gyri (*k* = 45,* X*/*Y*/*Z* = 22/ − 76/ − 14, *t* = 4.73, *p* = 0.019), in another small cluster in the rMFG (*k* = 6, *X*/*Y*/*Z* =  − 34/22/45, *t* = 4.40, *p* = 0.031) and left precentral gyrus (*k* = 1, *X*/*Y*/*Z* =  − 39/0/46, *t* = 4.18, *p* = 0.048) (all FDR-corrected *p*-values) (Fig. 2). Distress severity showed no positive or negative relationship with GMV after FDR correction for statistical significance.

### Moderating effects of PLE distress severity

The prefrontal VBM cluster showed two local maxima in the dorsolateral prefrontal cortex (DLPFC), indicating the middle (rMFG) and superior frontal gyri (rSFG). For *Delusional,* no effect was observed in either rMFG or rSFG model. A moderating effect of *Perceptual* in the rMFG was only significant at trend-level [unstandardized coefficient =  − 0.15, SE = 0.08, *t*(97) =  − 1.90, *p*_FDR_ = 0.060], while the overall significant model for the rSFG [*F*(6,97) = 22.06, *p* < 0.001, *R*^*2*^ = 0.52] showed a significant moderation of *Perceptual* distress scores ≥2.75 [unstandardised coefficient =  − 0.09, SE = 0.04, *t*(97) =  − 2.32, *p*_FDR_ = 0.044], with increased *Perceptual* distress resulting in decreased GM value (Table 3). Due to an overrepresentation of females, the additional nonsignificant moderating effect of sex on this pathway (i.e. PLE × Perceptual PLE distress × Sex interaction) was inspected with the PROCESS macro.

**Table 3:** Regression models

Dependent variable	Predictor	Perceptual PLE						Delusional PLE					
		*F*(6,97) = 22.06, *p* < 0.001, *R*^*2*^ = 0.52						*F*(6,97) = 19.17, *p* < 0.001, *R*^*2*^ = 0.51					
		Coefficient (SE)^a^	*t*	*p*	*p*_FDR_^b^	LLCI^c^	ULCI^c^	Coefficient (SE)^a^	*t*	*p*	*p*_FDR_^b^	LLCI^c^	ULCI^c^
Superior frontal gyrus	Intercept	2.85 (1.90)	1.50	0.137	0.274	− 0.92	6.62	2.80 (1.98)	1.41	0.161	0.245	− 1.14	6.73
	Sex	0.38 (0.33)	1.12	0.263	0.526	− 0.29	1.04	0.38 (0.34)	1.09	0.277	0.554	− 0.31	1.06
	Age	− 0.07 (0.02)	− 3.47	0.001	0.001	− 0.11	− 0.03	− 0.07 (0.02)	− 3.24	0.002	0.002	− 0.12	− 0.03
	TIV	0.01 (0.00)	8.34	5 × 10^–13^	5 × 10^–13^	0.01	0.01	0.01 (0.00)	8.09	2 × 10^–12^	2 × 10^–12^	0.01	0.01
	Total PLE	0.07 (0.10)	0.69	0.493	0.493	− 0.13	0.26	0.01 (0.13)	0.05	0.959	0.959	− 0.25	0.26
	Distress	0.40 (0.18)	2.23	0.028	0.056	0.04	0.76	0.14 (0.24)	0.61	0.544	0.544	− 0.33	0.62
	*PLE* × *distress*	− 0.09 (0.04)	− 2.32	0.022	0.044	− 0.17	− 0.01	− 0.02 (0.05)	− 0.47	0.640	0.640	− 0.13	0.08

	Predictor	Perceptual PLE						Delusional PLE					
		*F*(6,97) = 43.67, *p* < 0.001, *R*^*2*^ = 0.64						*F*(6,97) = 37.17, *p* < 0.001, *R*^*2*^ = 0.66					
		Coefficient (SE)^a^	*t*	*p*	*p*_FDR_^b^	LLCI^c^	ULCI^c^	Coefficient (SE)^a^	*t*	*p*	*p*_FDR_^b^	LLCI^c^	ULCI^c^
Middle frontal gyrus	Intercept	2.57 (2.78)	0.92	0.358	0.358	− 2.95	8.09	3.39 (2.90)	1.17	0.245	0.245	− 2.36	9.13
	Sex	− 0.18 (0.45)	− 0.39	0.700	0.700	− 1.08	0.72	− 0.26 (0.45)	− 0.57	0.572	0.572	− 1.15	0.64
	Age	− 0.11 (0.04)	− 3.28	0.001	0.001	− 0.18	− 0.05	− 0.13 (0.04)	− 3.59	0.001	0.002	− 0.20	− 0.06
	TIV	0.01 (0.00)	9.56	1 × 10^–15^	2 × 10^–15^	0.01	0.02	0.01 (0.00)	9.01	2 × 10^–14^	4 × 10^–14^	0.01	0.02
	Total PLE	0.19 (0.16)	1.21	0.231	0.462	− 0.12	0.51	0.40 (0.39)	1.04	0.303	0.303	− 0.37	1.17
	Distress	0.52 (0.31)	1.70	0.093	0.093	− 0.09	1.13	0.43 (0.51)	0.85	0.396	0.544	− 0.57	1.44
	*PLE* × *distress*	− 0.15 (0.08)	− 1.90	0.060	0.060	− 0.30	0.01	− 0.24 (0.31)	− 0.77	0.444	0.640	− 0.86	0.38

^a^SE = Cribari-Neto heteroskedasticity-consistent standard error

^b^*p*_FDR_ = *p-*value after false discovery rate (FDR) adjustment

^c^LLCI = 95% lower (LLCI) and upper (ULCI) limit confidence interval

## Discussion

The present study aimed to elucidate the relationship between brain structure and PLE in nonclinical subjects devoid of attenuated risk for psychosis. The results revealed a positive association between PLE and volume in right dorsolateral prefrontal, fusiform and occipital brain regions, which was not present for the distress severity scale. However, exploratory analysis of the whole right superior and middle frontal gyral volumes showed a modulating effect of distress severity.

The main finding of this study is that PLE applicable for psychosis risk screening are associated with neurobiological changes independent of UHR case–control status, conversion [61], and UHR phenotype heterogeneity (e.g., genetic risk deterioration syndrome, attenuated psychotic syndrome, brief limited intermittent psychotic symptoms) [62]. Correlates for subclinical PLE were detected in the right hemisphere. This differs from clinical findings in schizophrenia, showing either left lateral or bilateral GM reductions in the medial and superior temporal lobes [63, 64] and a linkage with severity of auditory hallucinations [65]. However, GM alterations in the right dorsolateral prefrontal cortex are also represented in studies of schizophrenia and diverse prodromal stages [63, 66–69]. Regional GM differences between healthy, genetic-high risk, and first-episode schizophrenia individuals also highlight genetic components [70]. Our significant regional findings align with some of those found in the genetic-high risk group in Chang et al.[70], such as larger volumes in rMFG and fusiform gyrus compared to healthy controls. Interestingly, a large genome-wide association study recently demonstrated shared genetic liability between PLE and multiple psychiatric conditions [71].

Magnitude of GMV loss shows some variability over disease progression [72], and progressive structural differences were also seen in reduced white matter growth in UHR adolescents [73]. Accelerated prefrontal GMV loss may indicate differential pathological processes at different neurodevelopmental stages in schizophrenia [74]. This would be in line with potentially non-linear patterns of brain structural changes dependent on transition and illness phase [75]. However, another comparison of CHR youths to controls could not confirm structural and cortical thickness differences regardless of later transition to psychosis [76]. In that study, the critical role of sample uniqueness, especially the absence of illicit drug use, including cannabis, are discussed. An extension of our design would be an exploration of the effect of illicit drug use on the observed PLE-brain structural relationship.

Contrary to predictions, we found a positive direction for the association between PLE and GMV. In the earlier analysis [39], positive schizotypal traits were associated with GMV reductions in superior and middle frontal gyri. Tract-based white matter and GMV analyses implicated alterations in fronto-striatal network regions in schizotypy. However, it remains speculative whether all schizotypy dimensions equally reflect neural deficits or vulnerability. The proximity between PLE and positive schizotypy is further supported by their anatomical overlap, however, PLE correlated with larger volumes in a prefrontal cluster. Together the findings from these two studies do not support a linear continuum ranging from the subclinical phenotypes to CHR and schizophrenia spectrum disorders [31, 77].

The right superior and middle frontal gyri, which are cortical correlates in CHR and transition status [26, 78], could imply modulation by intraindividual psychological factors that may convey vulnerability or resilience in nonclinical individuals, too. Larger DLPFC volumes may be explained by compensatory mechanisms, e.g., in response to upstream striatal alterations [77]. Compensatory processes were also proposed for larger precuneus and posterior cingulate cortex volumes in association with nonclinical psychosis proneness [34, 79, 80], despite volume reductions in the clinical spectrum being common [68, 81, 82]. In that case, larger regional prefrontal volumes at higher PLE levels, but reductions related to the interaction of overall PLE and distress severity of perceptual anomalies, may indicate attenuated protective features. This buffering explanation was earlier proposed by Meller et al. [49], showing that the association between positive schizotypy and larger striatal volume is decreased by general intelligence (a functional substrate of the frontal regions). Preservation of prefrontal functions and GMV [83] may be pivotal determinants of clinical deterioration and prevention. A comparison of brain developmental trajectories in resilient and non-resilient UHR youths found larger frontal volumes over time in the higher functioning group [27]. Resilience [84] may contribute to prefrontal cortical variation in nonclinical subjects as well. This would be in keeping with the notion that PLE are manifestations of the positive schizotypy dimension [43], which correlates with psychosis-relevant genotypes involved in dopamine regulation [45]. A specific effect for the perceptual PLE component also fits in with the striatal dopamine hypothesis underlying psychosis in schizophrenia and general psychosis proneness [47].

Additionally, the right occipital fusiform and lingual regions were positively associated with PLE. This finding in the occipitotemporal region indicates unique PLE correlates that were not present in multidimensional schizotypy. Involvement of the fusiform gyrus in perception and face recognition [85, 86], together with occipitotemporal GMV reductions in schizophrenia and psychosis [87–89], underpins deficits related to facial processing in the clinical spectrum [90]. One PQ-16 item (‘When I look at a person, or look at myself in a mirror, I have seen the face change right before my eyes’) may have been especially relevant to the diametrically opposed outcome in nonclinical individuals. Another study reported associations between positive PLE distress and precuneus volume, which were not present in trait psychosis proneness [38]. Our findings for a positive association for PLE load located in the dorsolateral cortical regions as opposed to parietal brain regions may be explained by differences between purely quantitative PLE levels, and measures relating to the qualitative burden of PLE. Failure to replicate precuneus correlates for PLE distress in the present study may be attributed to differing psychometric PLE measures related to different aspects of psychosis proneness. Nonetheless, they complement each other in that they underline the impact of perceiving positive symptoms as worrisome in brain regions implicated across the psychosis spectrum.

Some limitations of this study require evaluation. Although the present cohort consists of young adults, we must acknowledge that cross-sectional designs do not permit prediction of subsequent psychopathological development. Another inherent problem of studies with nonclinical designs is a non-normal PLE distribution [91]. Also, the size of the study cohort was limited, which might have hampered the detection of smaller effects. Adoption of instrument (long vs. short PQ versions), setting, and UHR enrichment are sources of detection threshold variability [13]. Self-reported PLE are poor measures of clinician-rated psychosis risk [15], and current recommendations state clinical CHR assessment should only be extended to those distressed by symptoms [1]. Note also that symptom dimensions were based on the assessment of item contents but require validation using factor analysis. This is especially recommended for positive items where the latent delusional or perceptual character is ambiguous. Brandizzi and colleagues’ [23] analysis of PQ-92 positive items yielded factors reflecting ‘perceptual abnormalities’, ‘bizarre experiences’, as well as ‘conceptual disorganisation and suspiciousness’ and ‘magical ideation’ also found in schizotypy. Additionally, Kotzalidis et al. [56] identified a four-factor solution, including a heterogeneous ‘functional’ dimension. While this provides options for replication using the extended versions of the Prodromal Questionnaire, the translation of these factors to the 16-item screening inventory seems unlikely.

While further replication in larger samples is warranted, our findings go beyond symptom-structure associations by showing the moderating impact of the distress dimension on the anatomical underpinnings of PLE. This posits a crucial distinction for future dimensional model studies as the marked distinction between (positive) subclinical symptoms with varying degrees of subjective impact is stressed. It is currently expected that neuroimaging studies will provide complementary tools for predicting transition to psychosis [92] and long-term clinical outcomes [93]. Our study supports these attempts by isolating the neurobiological uniqueness of PLE in the nonclinical part of the psychosis spectrum. We suggest that future investigations might also address the neurobiological characterisation of resilience in genotypes and phenotypes related to psychosis proneness.

## Data Availability

All original data are on record and accessible to inspection, but are not available publicly based on local and national data protection regulations.

## Code availability

All software used in the analyses is based on publicly available code.
